# Advances in Roles of miR-132 in the Nervous System

**DOI:** 10.3389/fphar.2017.00770

**Published:** 2017-10-25

**Authors:** Yun Qian, Jialin Song, Yuanming Ouyang, Qixin Han, Wei Chen, Xiaotian Zhao, Yangmei Xie, Yinghui Chen, Weien Yuan, Cunyi Fan

**Affiliations:** ^1^Shanghai Jiao Tong University Affiliated Sixth People’s Hospital, Shanghai, China; ^2^Shanghai Sixth People’s Hospital East Campus, Shanghai University of Medicine and Health, Shanghai, China; ^3^Renji Hospital, School of Medicine, Shanghai Jiao Tong University, Shanghai, China; ^4^School of Pharmacy, Shanghai Jiao Tong University, Shanghai, China; ^5^Department of Neurology, Jinshan Hospital, Fudan University, Shanghai, China

**Keywords:** miR-132, nervous system diseases, signaling pathways, axon growth, neural migration

## Abstract

miR-132 is an endogenous small RNA and controls post-transcriptional regulation of gene expression via controlled degradation of mRNA or transcription inhibition. In the nervous system, miR-132 is significant for regulating neuronal differentiation, maturation and functioning, and widely participates in axon growth, neural migration, and plasticity. The miR-132 is affected by factors like mRNA expression, functional redundancy, and signaling cascades. It targets multiple downstream molecules to influence physiological and pathological neuronal activities. MiR-132 can influence the pathogenesis of many diseases, especially in the nervous system. The dysregulation of miR-132 results in the occurrence and exacerbation of neural developmental, degenerative diseases, like Alzheimer’s disease, Parkinson’s disease and epilepsy, neural infection and psychiatric disorders including disturbance of consciousness, cognition and memory, depression and schizophrenia. Regulation of miR-132 expression relieves symptoms, alleviates severity and finally effects a cure. This review aims to discuss the clinical potentials of miR-132 in the nervous system.

## Introduction

MicroRNA (miRNA) is an endogenic RNA composed of 20 to 24 nucleotides, in combination with the 3′ untranslated region (3′UTR) to regulate the expression of gene transcription (Reschke and Henshall, 2015). Currently, around 1500 genes encoded by miRNAs have been confirmed in the human genome, regulating the expression of various mRNAs in different physiological and biological conditions. Among these miRNAs, miR-132 has been mentioned in many articles that illustrate its role in the neuronal development and functioning. Besides, its part in diverse diseases of the nervous system has not been elucidated clearly yet. miR-132 was initially found in nerve tissues of mice (Lagos-Quintana et al., 2001), later in human (Calin et al., 2004), zebrafish (Chen et al., 2005), and cow (Coutinho et al., 2007). Mature miR-132 sequences with a length of 22 base pairs (bp) are processed by precursor sequences with 66 bp in length (Coutinho et al., 2007). miR-132 in the human body is composed of two homologous miRNAs, hsa-miR-132-5p and hsa-miR-132-3p. The latter miRNA is a significant part of the miR-132/212 cluster, targets and decreases spinal AMPA receptor subunit GluA1 as a negative regulator of chronic neuropathic pain (Leinders et al., 2016). miR-132 has undergone conservative evolution in humans, rats, mice, apes, and other species, exhibiting the same sequence and structure (Sun et al., 2015).

Regulation of miR-132 in a biological process is critical to physiological balance among various systems in human bodies. It is influenced by several factors including expression levels of miR-132 and its mRNA target, overlapping functional redundancy, temporal patterns of miR-132 changes, signaling feedbacks and other control mechanisms, as well as miR-132 longevity with long-lasting effects on protein output (Ebert and Sharp, 2012). Once the balance is broken, dysregulation of miR-132 will lead to occurrence and exacerbation of multiple disorders from different systems, including the nervous system.

miR-132 represents a broad category of nerve regulatory networks in the dynamic regulation of neuronal differentiation, maturation and functioning, and participates in axon growth, neural migration, and plasticity. The dysfunction of miR-132 occurs widely in neural development, neurodegenerative diseases and psychological and mental disorders (**Table 1**). Regulation of miR-132 expression and downstream signaling cascades potentially relieves symptoms, alleviates severity and finally effects a cure.

**Table 1:** Relevance of miR-132 for various neuropathologies.

Neuropathology	Direction of expression change	Validated target	Outcomes for changes of miR-132 expression	*In vitro/in vivo*	Reference
Multiple sclerosis	Up-regulation	Sirtuin-1	Induced the sharp increase in the proinflammatory factors LT and TNF-α	*In vitro*, human blood	Miyazaki et al., 2014
Alzheimer’s disease	Down-regulation	PTEN, FOXO3a	Caused TUNEL-positive neuron accumulation and caspase-dependent apoptosis	*In vitro*, SD rat embros/*in vivo* rat	Luikart et al., 2011; Wong et al., 2013
Parkinson’s disease	Up-regulation	Nurr1	Affected the midbrain dopamine projections with the disruption of dopamine transmission in the basal ganglia	*In vitro*, rat brain/*In vitro*, mice	Decressac et al., 2012; Lungu et al., 2013
Progressive supranuclear palsy	Down-regulation	PTBP2	Caused abnormal intracellular deposits of the microtubule-associated protein tau	*In vitro*, human brain	Smith et al., 2011
Virus infection	Up-regulation	FOXP1, FOXP2	induced nervous system complications, such as encephalitis in VZV	*In vitro*, human serum	Qi et al., 2014
Prion diseases	Up-regulation	NMDA	blocked synaptic N-methyl-D-aspartate (N-methyl-D-aspartic acid, NMDA) receptor signaling pathways to suppress the protection function	*In vitro*, mice	Majer et al., 2012
Parasite infection	Up-regulation	DRD1, DRD5, MAOA	Increased neuronal spine density but impaired novel object recognition and spatial memory	*In vitro*, human neuroepithelioma cells/*in vitro*	Hansen et al., 2010; Xiao et al., 2014
Epilepsy	Up-regulation	SOX11	Aggravated neuronal damage after status epilepticus attacks	*In vivo*, SD rat/*in vivo*, mice	Jimenez-Mateos et al., 2011; Peng et al., 2013
Disturbance of consciousness and cognition	Up-regulation	AChE, p250GAP	Imbalanced excessive axonal budding-related nerve damage	*In vivo*, mice	Hansen et al., 2013
Memory disturbance	Up-regulation	Rac1, p250GAP	Disregulated the development for synaptic morphological structure of dendritic spines	*In vitro*, mouse neuron cells	Kempf et al., 2014
Depression	Down-regulation	Not mentioned	severe damage to the loop transformation of excitatory synapses of new neurons in the adult brain	*In vivo*, mice	Yi et al., 2014
Schizophrenia	Up-regulation	Dnmt3a, Dpysl3, NR1 and GAD67	Disturbed the pattern of gene expression in the PFC during adolescent neurodevelopment	*In vivo*, mice/*in vivo*, mice	Miller et al., 2012; Balu et al., 2013


## Physiological Function of miR-132 in the Nervous System

### Neural Growth and Migration

The physiological involvement of miR-132 in the process of neural growth and migration requires multiple interactions with other cells. Radial glial progenitors, regulated by the Notch signaling pathway, have significant implications in the neural growth and patterning of spinal cord. Salta and colleagues identified a bidirectional regulatory circuit in a temporal manner between miR-132 and its target Ctbp2 (C-terminal binding protein 2). This circuit dynamically contributes to the transduction of Notch signaling and its downstream effects on glial progenitors. During early stage of embryogenesis, increasing Ctbp2 represses transcriptional level of Sirtuin-1 to initiate Notch signaling cascade, resulting in the progressive accumulation of the glial progenitor cells. With significant increase of Ctbp2, miR-132 expression becomes much higher and suppresses Ctbp2 expression after reaching a certain threshold. These processes contribute to a bidirectional Notch signaling pathway in Gfap+ glial progenitors (Salta et al., 2014). Moreover, block of miR132-mediated repression contributed to the overexpression of MeCP2 and BDNF (brain-derived neurotrophic factor) levels *in vivo* (Klein et al., 2007).

Besides, miR-132 impacts on the neural migration in mammals. By profiling gene expression and knocking out miR-132 in the mice dorsal root ganglion, Clovis and colleagues identified the ectopic expression of a new form of transcription factor Foxp2 in cortex projecting neurons. Therefore, the group believes knocking out miR-132 leads to a reduction of Foxp2 expression, a delay of neurite outgrowth and blockage of radial migration for neural cells. Moreover, proliferation and branching is precisely regulated by a coordinated effort of miR-9 and miR-132 for Foxp2 derived 3′UTR (Clovis et al., 2012).

### Cell Differentiation

The relationship between miRNAs and cell differentiation has long been studied. miR-132 directly targets the expression of nuclear receptor related protein 1 (Nurr1) to induce the differentiation of embryonic stem cells (ESCs) into dopamine neurons. In the mouse ESCs, Yang and colleagues noticed that endogenous miR-132 expression was directly down-regulated by the antisense oligonucleotide, thereby promoting the cell differentiation of tyrosine hydroxylase-positive nerve cells. Furthermore, the antisense oligonucleotide is even capable of reversing the neuronal differentiation of the miR-132-mediated inhibitory effect (Yang D. et al., 2012). Besides, Yoshimura and colleagues pointed out that silencing miR-132 or suppressing ERK1/2 signaling pathway activation led to the increased expression of a variety of synaptic proteins, including PSD-95, GluR1 and synapsin I (Yoshimura et al., 2016). Therefore, miR-132 can be regarded as a negative regulator of synapse maturation and cell differentiation.

### Neural Plasticity

Apart from cell proliferation and differentiation, miR-132 is also implicated in the process of modulation of specific neuronal and immune function. Remenyi and colleagues found that in miR-132/212 knockout mouse models, miR-132 could affect cortex synaptic transmission and plasticity (Remenyi et al., 2013). Developing axons can localize protein synthesis. In the axon, miR-132 is present at high concentrations and regulates axonal growth through the target gene Rasa1. Hancock and colleagues proposed two possible mechanisms for the regulation of the activity of miR-132: potential axonal guidance and the temporal regulation associated with the developmental stage (Hancock et al., 2014). Another mechanism for regulation of miR-132 is raised by Cheng and colleagues. They believe that miR-132 attenuates the entraining effects of light, induced by photic stimulation via a MAPK/CREB signaling pathway (Cheng et al., 2007).

**FIGURE 1:**
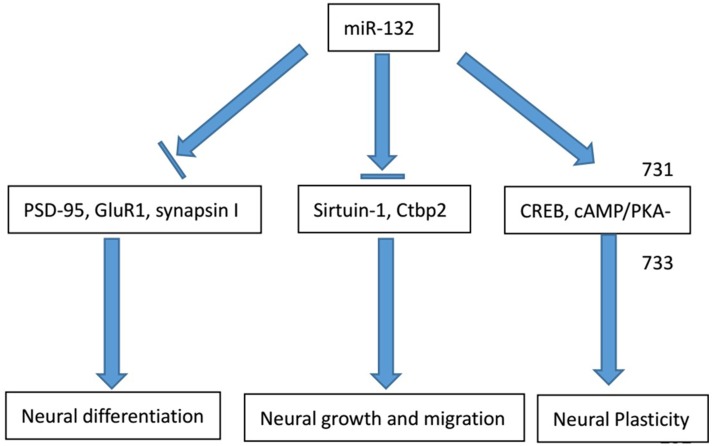
Major physiological functions of miR-132 in the nervous system. The physiological involvement of miR-132 in the process of neural growth and migration, cell differentiation and neural plasticity and major regulator genes in the process are listed above.

Apart from miR-132 knock out, silencing other molecules can also contribute to negative regulation of neural plasticity together with miR-132. Luteolin, a type of flavonoid, acts as a nerve nutrition factor. By knocking out luteolin, Lin and colleagues demonstrated that the inhibition of axon growth with the combined effect of miR-132 and luteolin, suggesting miR-132 might regulate nerve axon formation. They think that luteolin can activate the cAMP response element binding protein via phosphorylation to improve the miR-132 expression level and axon growth. According to the findings, luteolin therapy can enhance the level of ERK phosphorylation and PKA activity by inducing miR-132 expression and promoting axonal growth (Lin et al., 2012). Based on the researches above, we conclude the main roles of miR-132 in neurological conditions (**Figure 1**).

## The Relationship Between miR-132 and Neurological Disorders

### Neurodegenerative Diseases

#### Multiple Sclerosis

Multiple sclerosis (MS) is a demyelinating disorder with damaged insulating covers of neurons in the brain and spinal cord. This damage destroyed partial ability of communication in the nervous system, resulting in plenty of signs and symptoms, involving mental, physical, and sometimes psychiatric problems (Compston and Coles, 2002). Miyazaki and colleagues found a significant rise in miR-132 expression in B cells accompanied with the disease onset. Compared with the healthy group, dual B-cell antigen receptor (BCR)+CD40 stimulation considerably increased the expression of lymphotoxin (LT) and TNF-α, along with a significant increase in miR-132 (*p* = 0.0143). MiR-132 does not directly target LT and TNF-α, indicating an indirect pathway. (Miyazaki et al., 2014; **Table 1**).

Nakahama and colleagues used Sirtuin-1, a negative regulator of miR-132 to study the underlying mechanism. Sirtuin-1, member of the histone acetylation enzyme III family, plays an important role in aging, metabolism, tumor formation, inflammation, and a series of immune reactions. Sirtuin-1 possibly inhibits the transcription factors NF-kB and AP-1 in BCR signaling pathways, thus inhibiting the downstream LT and TNF-α expression. Excitation of Sirtuin-1 is regulated by resveratrol, a small molecule that can pass the blood brain barrier and suppress pro-inflammatory cellular responses in the peripheral circulation and target organs (Nakahama et al., 2013). Yeung and colleagues also proposed that Sirtuin-1 could reduce nerve damage and regulate nerve protection in muscle atrophy sclerosis and Alzheimer’s disease (Yeung et al., 2004). Sirtuin-1 down-regulates miR-132 expression and regulates downstream targets, offering a meaningful alternative therapy in MS treatment.

#### Alzheimer’s Disease

Alzheimer’s disease (AD) is a neurodegenerative disease with a progressive development style and one of the most common types of dementia. In 30 years, it is predicted that there will be over 100 million AD patients. The synapses and neurons are lost at a significant rate in the basal forebrain, cortex and hippocampus in these patients, resulting in cognitive decline and other symptoms. The abnormal expression of miRNA regulates major AD-related genes, such as APP and BACE1, affects Aβ cytotoxicity and neural death, and even marks characteristic changes in disease initiation and progression (Fang et al., 2012). Wong and colleagues found that the decrease of miR-132/212 could be detected at the onset of AD, and it became especially distinct at the late stage. In primary cultured neurons, the inhibition of miR-132/212 expression will lead to TUNEL-positive neuron accumulation and caspase-dependent apoptosis. They prove that PTEN and FOXO3a are two direct targets and suppress the AKT signaling pathway (Wong et al., 2013; **Table 1**). The inhibition of either miR-132 or miR-212 will eliminate the inhibition of PTEN and FOXO3a and trigger programmed death.

Similarly, the miR-132/212 cluster in late-onset Alzheimer’s disease (LOAD) appears to be in significant decline. miR-132-3p exhibits different levels of decreased expression in three different parts – the hypothalamus, the frontal cortex and medial temporal area (Luikart et al., 2011). Through the analysis of the LOAD samples from the hypothalamus, expression levels are found to be promoted in glial-rich miR-142-3p, miR-150-5p and miR-223-3p. However, these miRNAs can only be detected in the middle-late stage, so they only explain the loss of neurons of neurodegenerative diseases in late stages. miR-132-3p is dysregulated at early courses of the disease, especially before neuronal loss occurs. Therefore, miR-132 is closely related to LOAD in different stages (Magill et al., 2010). miR-132 with a high synaptic activity and plasticity, regulates neural outgrowth under the effect of nerve growth factors. Moreover, it controls the combination of newborn neurons in adult hypothalamus and dentate gyrus, and shapes synapse formation and plasticity in the visual cortex. miR-132 has many molecular targets, such as Tau, EP300, Sirtuin-1 and FOXO1a (Min et al., 2010). In the AD samples of the hypothalamus, the protein content of FOXO1a obviously increased (Lau et al., 2013). By examining the expression of different downstream targets of miR-132, we are provided with a new way to predict disease progression.

#### Parkinson’s Disease

Parkinson’s disease (PD) is closely associated with age and the second most common neurodegenerative disorder after Alzheimer’s disease. It attacks 1–2% people aged 60 and above (Yoshimura et al., 2016). The neurodegenerative process in PD affects the midbrain dopamine projections from the substantia nigra with the disruption of dopamine transmission in the motor loop of the basal ganglia. Lungu and colleagues developed a spontaneous autosomal recessive rat model for neurodegeneration and found a decreased expression of BDNF in the midbrain and plasma. Increased synaptic nucleoprotein α and decreased Nurr1 transcription factor lead to a significant increase in the miR-132 level (Lungu et al., 2013; **Table 1**). Decressac and colleagues showed the downstream target of miR-132, Nurr1 nucleoprotein, has an anti- toxic effect on nucleoprotein α (Decressac et al., 2012; **Table 1**). Therefore, Nurr1 is the most important transcription factor for midbrain dopamine neurons to survive and to maintain their differentiation ability, serving as a potential new target for nerve protection.

#### Tauopathies: Progressive Supranuclear Palsy

Tauopathies account for a major group of neurological and movement diseases with a significant characteristics that its main cause, protein tau exhibits abnormal deposits in the cells. The miRNA expression information from patients who had progressive supranuclear palsy (PSP), a primary 4R-tau disorder was comprehensively analyzed, which displayed a downregulation of miR-132. Moreover, miR-132 can target polypyrimidine tract binding protein 2 (PTBP2) directly, which is a major neuronal splicing factor and increased in protein level from PSP patients. Overexpressed miR-132 or silenced PTBP2 can influence endogenous 4R:3R-tau proportion in neurons (Smith et al., 2011; **Table 1**).

### Epilepsy

Epilepsy is a chronically progressive neurological disease originating from unusual neuronal discharges in the brain. There are nearly 50 million epileptic patients in the world, which puts heavy burden on societies. The present anti-epileptic therapy aims at inhibiting convulsant or epileptic effects. They can control the frequency of seizure attacks, but attempts on revealing the underlying pathophysiology of epilepsy show little progress. Nudelman and colleagues found that pilocarpine induced epilepsy led to a significant upregulation of primiR-132 and mature miR-132. miR-132 in patients with epilepsy tolerance presents a significant ability to react to different stimuli (Nudelman et al., 2010). Studies on the functional sequence of miR-132 showed that before status epilepticus occurs, miR-132 antagomirs, which can inhibit expression of miR-132, does not reduce epilepsy risks but can significantly reduce the hippocampal nerve damage, which confirms that the excessive expression of miR-132 aggravates neuronal damage after status epilepticus attacks (Peng et al., 2013; **Table 1**). Researchers also found that levels of miR-132 in the acute and chronic phases were significantly increased. In the latency stage, the expression of miR-132 was still at a high level (Jimenez-Mateos et al., 2011; **Table 1**). Moreover, micro-injection of the antagomirs can significantly decrease miR-132 and reduce mortality caused by the abnormal epileptic discharge of neurons. The increase of miR-132 in the latency stage of epilepsy provides new insight into the prediction and control of the disease.

The role of miR-132 in the pathogenesis of epilepsy also involves the participation of other genes and factors. In a focal medial temporal lobe epilepsy model, the 3′UTR binding sites of the transcription factor SOX11 were found to be connected with miR-132-3p to regulate the branching and bifurcation of newborn neurons. Using a status epilepticus hippocampus neuron model, Lei and colleagues found that miR-132 and BDNF mRNA increased significantly. Meanwhile changes in epileptic discharge were noticed as well. BDNF preprocessing catabatic TrkB receptor activation can partially reduce high frequency discharge, but the excessive expression of miR-132 deteriorates the symptoms of epileptic discharge, proving the control of miR-132 on the BDNF/TrkB signaling pathway as an important mechanism of epilepsy (Xiang et al., 2015).

### Neural Infection

#### Virus and Prion Infection

Varicella, also called chickenpox, is a highly contagious disease caused by varicella zoster virus (VZV). As novel and non-invasive indicators for the diagnosis of contagious disease, circulating miRNAs have been intensively researched. Qi and colleagues found that miR-132 could distinguish varicella patients from healthy controls and other microbial infections with moderate sensitivity and specificity (Qi et al., 2014; **Table 1**).

Prion disease, a kind of deadly disease can cause neurodegeneration and affect mammals. The underlying pathogenesis is probably that normal cellular prion protein, (PrPC) is turned into the infectious type, PrPSc (Scrapie prion protein). Although this happens over a long preclinical latency stage, compromised nerve function is noticed at asymptomatic stages. Particularly, in animal models of prion disease, loss of synaptic function occurs before the onset of clinical symptoms. Majer and colleagues separated hippocampal CA1 neurons, determined the preclinical transcriptional response, and found the abnormal transcription of miR-132-3p blocked synaptic *N*-methyl-D-aspartate (*N*-methyl-D-aspartic acid, NMDA) receptor signaling pathways to suppress the protection function (Majer et al., 2012; **Table 1**). miR-132 plays an indirect role in the pathogenesis of prion disease and target management of miR-132 seems to be a promising method for prevention.

#### Parasite Infection

*Toxoplasma gondii* (*T. gondii*) is a highly infectious parasite to living creatures. It is discovered that *T. gondii* impacts intermediate hosts on their behavioral changes as long as it progresses to a late stage, distinguished by the parasite cysts existing in the brain. Li and colleagues found that miR-132, was the only miRNA that was apparently increased in all three prototype Toxoplasma strains. They predicted the potential target and identified 20 genes and the strongest associated pathway dopamine receptor signaling cascade (Li et al., 2015; **Table 1**).

In chronically infected mice models, it was discovered that miR-132 overexpression upregulated nerve spine density in transgenic mice at the cost of recognition and memory functions (Xiao et al., 2014; **Table 1**). Wang and colleagues reported that when the expression of miR-132 was decreased in hippocampus, their ability for tracing fear memory was compromised at the same time. Therefore, in *Toxoplasma gondii* infection, the memory of infected patients will be impaired whether miR-132 is increased or decreased. The detection of five brain regions showed that women were infected in the hypothalamus, hippocampus, striatum and brainstem. However, men were infected only in the hippocampus. This confirms that sex is an important factor in the severity of disease (Hansen et al., 2010; **Table 1**). In spite of the regulatory ability of BDNF for miR-132 in neurons, it is not correlated in the brains of individuals with Toxoplasma infection. Wanet and colleagues found that other transcription factors, such as CREB and REST, may affect the expression level of miR-132 in patients with chronic infection (Wanet et al., 2012).

### Psychological Diseases

#### Disturbance of Consciousness and Cognition

Apart from the involvement in the neurologic diseases, miR-132 also deeply affects the psychological changes and formation of mental problems. In three different stress rat models, Shaltiel and colleagues found long-term stress can significantly improve the individual differences and expression levels of hippocampus miR-132. At the same time, the expression for two miR-132 targets AChE and GTP enzyme agonist p250GAP is restrained (Shaltiel et al., 2013). In a foot shock model, knocking out AChE significantly restricted the increase of miR-132. The results confirmed that the hippocampus miR-132 mediated the sustainability of the cholinergic signaling pathway, which was interrupted by AChE overexpression (Shaltiel et al., 2013). The inhibition of AChE will hinder cognitive defects stimulated by the plantar electric shocks, which indicates that miR-132 regulates the process of cognitive impairment after stress (Wayman et al., 2008). Under the AChE glucocorticoid reactive component activation, the ACh level drops and suppresses the CREB-induced transcription for miR-132, later achieving balance again (Vo et al., 2005). The inhibition of cortical neuron p250GAP will improve axial budding and neuronal activity, the GABAα inhibitor dicentrine can induce miR-132 transcription and reduce p250GAP expression levels to further promote axonal growth (Impey et al., 2010). These findings confirm the long-term double inhibition of AChE and p250GAP will balance excessive axonal budding-related nerve damages and become an important compensatory mechanism.

Hansen and colleagues showed miR-132 expression was upregulated prominently via a spatial memory mission by 1.5 folds, in excitatory hippocamal layers confirming the positive role of miR-132 in cognitive capacity (Hansen et al., 2013; **Table 1**).

#### Memory Disturbance

Hippocampal miR-132 is vital to the formation of trace fear memory. Mature miR-132 was up-regulated and reached a peak expression in 30 min with trace fear conditioning (TFC) and returned to a normal value after 2 h. The above information demonstrated the transient, expression of miR-132 was induced by activity (Lambert et al., 2010). The increased level of hippocampus miR-132 assists in memory regulation in dendritic spines and synaptic transmission. However, the excessive expression of miR-132 in transgenic mice, can increase the dendritic spine density but significantly damage the cognition of spatial memory (Wang et al., 2013). This indicates that proper miR-132 expression promotes good memory formation, and the lack or overexpression of miR-132 will damage cognitive function.

Besides, stimuli like high-dose ionizing radiation lead to severe learning and memory impairments. Kempf and colleagues observed radiation-induced reduction of Rac1 and miR-132 inhibits the GTPase-activating protein p250GAP to regulate Rac1 activity. In the irradiated hippocampus and cortex, the researchers observed similar changes in the signaling cascades compared with *in vitro* results. The reduced expression of miR-132 and Rac1 led to up-regulation in hippocampal cofilin which contributes to correct learning and memory for its necessity of correct spine and synapse morphology (Kempf et al., 2014; **Table 1**). This process is essential for the correct development of the synaptic morphological structure of dendritic spines to ensure proper memory and cognitive function.

#### Depression

Depression has become an increasingly prevalent health problem throughout the world. Yi and colleagues found that single anti-depression therapy has a poor effect in 30% of depression patients. miR-132 can improve nerve growth, synaptic function, and cognition and offers several antidepressant effects (Yi et al., 2014; **Table 1**). Natural Extracts compose of a major part in depression treatment. Oleanolic acid is a pentacyclic terpene that can secrete serotonin and inhibit ischemic brain damage (Yang Y.C. et al., 2012). BDNF and TrkB are closely related to neurotransmitter release and postsynaptic signals and participate in the antidepressant response of oleanolic acid. Researchers silenced miR-132 expression to inhibit oleanolic acid-mediated behavior improvement and nerve nutritional signal transduction pathways. Oleanolic acid regulates and inhibits the signal transduction pathway and neuron proliferation of chronic stress injury through hypothalamus TrkB receptor-mediated BDNF-ERK after CREB miR-132 (Luikart et al., 2011; **Table 1**). Previous studies have reported that BDNF can increase cortical cultivation and miR-132 expression levels of dopamine neurons (Numakawa et al., 2011a,b), which proves a great potential in antidepressant treatment.

### Schizophrenia

Schizophrenia is comprised of affective, cognitive, neuromorphological, and molecular abnormalities that originate from a problematic neurodevelopment. Accurate diagnosis of this disease seems to be a priority for early control and management. Yu and colleagues used dizocilpine to create a schizophrenia-like rat model and found that decrease of miR-132 in peripheral blood of 105 schizophrenia controls compared to 130 healthy controls. Besides, they also proved an apparent increase of miR-132 after risperidone treatment compared to that before treatment, further indicating the diagnostic role for miR-132 in schizophrenia (Yu et al., 2015).

As for the primary mechanism for schizophrenia onset, the dysregulation through NMDA/AMPA receptor-related signaling pathway may potentially disrupt cognitive development. Miller and colleagues found that miR-132 contributed to activity-oriented synaptic plasticity via NMDARs’ involvement in the positive feedback loop. A four-fold upregulation was noticed in miR-132 expression in the prefrontal cortex(PFC) in 3 weeks, after birth, a period with large quantity of synaptic modulation, via NMDAR signaling. Decreased miR-132 expression and the abnormal expression of Dpysl3 could affect synaptic activity and outgrowth negatively (Miller et al., 2012; **Table 1**). The above conclusions indicated that miR-132 was significant for gene expression mode in the PFC neurodevelopment.

The maturation of prefrontal GABAergic interneurons, which aim at excitatory nerve cells, is the second major process during late neuron development. With the possible functions of miR-132 in the initiation of NMDAR cascade taken into account, dysregulated miR-132 expression may interrupt the maturation of GABA interneurons at a molecular level where decreased GAD67 was encoded by GAD1 gene and synthesized by GABA (Balu et al., 2013; **Table 1**).

## Interrelationship Between miR-132 and miR-212 in the Nervous System

miR-212, categorized to the same cluster as miR-132, also serves as a significant gene in the process of neuronal development in both physiological and pathological conditions. Close sequences and identical seeding region are noticed in miR-132 and miR-212, indicating their potential targeting the same mRNAs. When it comes to the role in neurologic diseases, a down-regulation of miR-212 is detected in anencephaly (Zhang et al., 2010). Besides, in the prefrontal cortex of individuals, the dysfunction of miR-132 and miR-212 is greatly influenced by psychological diseases (Kim et al., 2010). Moreover, down-regulation is detected in miR-132 and miR-212expression in the α-synuclein overexpressed tissue, which is a classic stereotype of PD (Gillardon et al., 2008). The interrelationship between miR-132 and miR-212 deserves further investigation and attention as to their promising roles in the physiological and pathological regulation and functioning in the nervous system.

## Discussion

Recently, microRNA (miR) modulators in neuronal processes convey diagnostic and therapeutic implications for regulating miR levels in many neurological diseases (Glass et al., 2010). As one of the most significant neural-related miRNAs, miR-132 mainly targets transcription factors and directly or indirectly affects neural signaling to the central and peripheral nervous systems. From the bidirectional regulatory circuit between miR-132 and Ctbp2 in neural migration, to cAMP/PKA- and ERK dependent CREB signaling cascade in neural modification, changing miR-132 expression manipulates neural signaling transduction and results in physiological activities. Moreover, miR-132 is considered to play a significant role in neural diseases, including caspase-dependent apoptosis, synaptic signaling blockage and excessive axonal damages, and to activate or inhibit downstream cascades, resulting in irreversible devastation in humans. Its upregulation or downregulation in some nerve tissues significantly affects normal neuron functions and activity. Besides, at different stages of neural diseases, the dysregulation of miR-132 could be regulated to different directions under many transcriptional factors. Therefore, miRNA target therapy and diagnosis pose a significant implication in various situations. The major advantages of miR-132 in therapeutic and diagnostic applications lie in its accuracy to track down signaling transduction in a series of neurological disorders and in its ability to multi-directionally regulate message feedback circuits. Moreover, its significance also enlightens us to improve drug development of target therapy, with the help of miRNA mimics and miRNA antagonists that function as regulators of important communication cascades. For now, the mechanism of miR-132’s regulation of downstream targets is not completely understood because the present research is limited by brain blood barrier and thus it is difficult to study miR-132 in nanomedicine transport in brain diseases. To overcome this problem, we need to further our investigation in the future.

Limited research has been carried out on the miR-132 physiological functions related to peripheral nervous system disorders. Yao and colleagues found miR-132 expression increased after hypoxia dependent sciatic nerve injury. The overexpression of miR-132 in Schwann cells significantly promoted cell migration, and the injection of a miR-132 agonist into a sciatic nerve lesion in mouse models accelerated the migration of the distal Schwann cells proximally (Yao et al., 2016). However, the molecular mechanism and the main signaling pathways have not been fully elucidated. miR-132 is an important neural-related miRNA, which promotes nerve growth, migration and differentiation in the central nervous system and peripheral nervous system. Without the intervention of the blood brain barrier, miR-132 is directly involved in the various complex physiological phenomena that regulate the progress of peripheral neuropathy through similar feedback as is in the hippocampus and thalamus. As an outrageously important neural-related indicator, miR-132 significantly contributes to the control of the neural network. It provides new opportunities for clinical diagnosis and treatment of central and peripheral nervous system diseases. Ongoing research is destined to transform our understanding of disease mechanism and future treatment clinically. Identifying new factors associated with the inhibition of nerve regeneration and providing more molecular targets for intervention will have a profound effect on miRNA research in the future.

## Author Contributions

YQ, JS, YO, QH, WC, XZ, YX, YC, CF, and WY participated in its design, searched databases, extracted and assessed studies and helped to draft the manuscript. WY conceived the initial idea and the conceptualization, participated in the data extraction and analysis. YC, CF, and WY revised the manuscript. All authors read and approved the final manuscript.

## Conflict of Interest Statement

The authors declare that the research was conducted in the absence of any commercial or financial relationships that could be construed as a potential conflict of interest.

## References

[B1] Balu D. T. Li Y. Puhl M. D. Benneyworth M. A. Basu A. C. Takagi S. (2013). Multiple risk pathways for schizophrenia converge in serine racemase knockout mice, a mouse model of NMDA receptor hypofunction. *Proc. Natl. Acad. Sci. U.S.A.* 110 E2400–E2409. 10.1073/pnas.1304308110 23729812 PMC3696825

[B2] Calin G. A. Sevignani C. Dumitru C. D. Hyslop T. Noch E. Yendamuri S. (2004). Human microRNA genes are frequently located at fragile sites and genomic regions involved in cancers. *Proc. Natl. Acad. Sci. U.S.A.* 101 2999–3004. 10.1073/pnas.0307323101 14973191 PMC365734

[B3] Chen P. Y. Manninga H. Slanchev K. Chien M. Russo J. J. Ju J. (2005). The developmental miRNA profiles of zebrafish as determined by small RNA cloning. *Genes Dev.* 19 1288–1293. 10.1101/gad.1310605 15937218 PMC1142552

[B4] Cheng H. Y. Papp J. W. Varlamova O. Dziema H. Russell B. Curfman J. P. (2007). microRNA modulation of circadian-clock period and entrainment. *Neuron* 54 813–829. 10.1016/j.neuron.2007.05.017 17553428 PMC2590749

[B5] Clovis Y. M. Enard W. Marinaro F. Huttner W. B. De-Pietri T. D. (2012). Convergent repression of Foxp2 3’UTR by miR-9 and miR-132 in embryonic mouse neocortex: implications for radial migration of neurons. *Development* 139 3332–3342. 10.1242/dev.078063 22874921

[B6] Compston A. Coles A. (2002). Multiple sclerosis. *Lancet* 359 1221–1231. 10.1016/S0140-6736(02)08220-X 11955556

[B7] Coutinho L. L. Matukumalli L. K. Sonstegard T. S. Van-Tassell C. P. Gasbarre L. C. Capuco A. V. (2007). Discovery and profiling of bovine microRNAs from immune-related and embryonic tissues. *Physiol. Genomics* 29 35–43. 10.1152/physiolgenomics.00081.2006 17105755

[B8] Decressac M. Kadkhodaei B. Mattsson B. Laguna A. Perlmann T. Björklund A. (2012). α-Synuclein-induced down-regulation of Nurr1 disrupts GDNF signaling in nigral dopamine neurons. *Sci. Transl. Med.* 4:163ra156. 10.1126/scitranslmed.3004676 23220632

[B9] Ebert M. S. Sharp P. A. (2012). Roles for microRNAs in conferring robustness to biological processes. *Cell* 149 515–524. 10.1016/j.cell.2012.04.005 22541426 PMC3351105

[B10] Fang M. Wang J. Zhang X. Geng Y. Hu Z. Rudd J. A. (2012). The miR-124 regulates the expression of BACE1/beta secretase correlated with cell death in Alzheimer’s disease. *Toxicol. Lett.* 209 94–105. 10.1016/j.toxlet.2011.11.032 22178568

[B11] Gillardon F. Mack M. Rist W. Schnack C. Lenter M. Hildebrandt T. (2008). MicroRNA and proteome expression profiling in early-symptomatic alpha-synuclein(A30P)- transgenic mice. *Proteomics Clin. Appl.* 2 697–705. 10.1002/prca.200780025 21136867

[B12] Glass C. K. Saijo K. Winner B. Marchetto M. C. Gage F. H. (2010). Mechanisms underlying inflammation in neurodegeneration. *Cell* 140 k918–934. 10.1016/j.cell.2010.02.016 20303880 PMC2873093

[B13] Hancock M. L. Preitner N. Quan J. Flanagan J. G. (2014). MicroRNA-132 is enriched in developing axons, locally regulates Rasa1 mRNA, and promotes axon extension. *J. Neurosci.* 34 66–78. 10.1523/JNEUROSCI.3371-13.2014 24381269 PMC3866495

[B14] Hansen K. F. Karelina K. Sakamoto K. Wayman G. A. Impey S. Obrietan K. (2013). MiRNA-132: a dynamic regulator of cognitive capacity. *Brain Struct. Funct.* 218 817–831. 10.1007/s00429-012-0431-4 22706759 PMC3508255

[B15] Hansen K. F. Sakamoto K. Wayman G. A. Impey S. Obrietan K. (2010). Transgenic miR132 alters neuronal spine density and impairs novel object recognition memory. *PLOS ONE* 5:e15497. 10.1371/journal.pone.0015497 21124738 PMC2993964

[B16] Impey S. Davare M. Lesiak A. Fortin D. Ando H. Varlamova O. (2010). An activity-induced microRNA controls dendritic spine formation by regulating Rac1-PAK signaling. *Mol. Cell Neurosci.* 43 146–156. 10.1016/j.mcn.2009.10.005 19850129 PMC2818337

[B17] Jimenez-Mateos E. M. Bray I. Sanz-Rodriguez A. Engel T. McKiernan R. C. Mouri G. (2011). MiRNA expression profile after status epilepticus and hippocampal neuroprotection by targeting miR-132. *Am. J. Pathol.* 179 2519–2532. 10.1016/j.ajpath.2011.07.036 21945804 PMC3204080

[B18] Kempf S. J. Buratovic S. von-Toerne C. Moertl S. Stenerlöw B. Hauck S. M. (2014). Ionising radiation immediately impairs synaptic plasticity-associated cytoskeletal signalling pathways in HT22 cells and in mouse brain: an In Vitro / In Vivo comparison study. *PLOS ONE* 9:e110464. 10.1371/journal.pone.0110464 25329592 PMC4203799

[B19] Kim A. H. Reimers M. Maher B. Williamson V. McMichael O. McClay J. L. (2010). MicroRNA expression profiling in the prefrontal cortex of individuals affected with schizophrenia and bipolar disorders. *Schizophr. Res.* 124 183–191. 10.1016/j.schres.2010.07.002 20675101 PMC4373420

[B20] Klein M. E. Lioy D. T. Ma L. Impey S. Mandel G. Goodman R. H. (2007). Homeostatic regulation of MeCP2 expression by a CREB-induced microRNA. *Nat. Neurosci.* 12 1513–1514. 10.1038/nn2010 17994015

[B21] Lagos-Quintana M. Rauhut R. Lendeckel W. Tuschl T. (2001). Identification of novel genes coding for small expressed RNAs. *Science* 294 853–858. 10.1126/science.1064921 11679670

[B22] Lambert T. J. Storm D. R. Sullivan J. M. (2010). MicroRNA132 modulates short-term synaptic plasticity but not basal release probability in hippocampal neurons. *PLOS ONE* 5:e15182. 10.1371/journal.pone.0015182 21206919 PMC3012071

[B23] Lau P. Bossers K. Janky R. Salta E. Frigerio C. S. Barbash S. (2013). Alteration of the microRNA network during the progression of Alzheimer’s disease. *EMBO Mol. Med.* 5 1613–1634. 10.1002/emmm.201201974 24014289 PMC3799583

[B24] Leinders M. Üçeyler N. Pritchard R. A. Sommer C. Sorkin L. S. (2016). Increased miR-132-3p expression is associated with chronic neuropathic pain. *Exp. Neurol.* 283 276–286. 10.1016/j.expneurol.2016.06.025 27349406 PMC4992589

[B25] Li Y. E. Kannan G. Pletnikov M. V. Yolken R. H. Xiao J. (2015). Chronic infection of *Toxoplasma gondii* downregulates miR-132 expression in multiple brain regions in a sex-dependent manner. *Parasitology.* 142 623–632. 10.1017/S003118201400167X 25351997 PMC4428143

[B26] Lin L. F. Chiu S. P. Wu M. J. Chen P. Y. Yen J. H. (2012). Luteolin induces microRNA-132 expression and modulates neurite outgrowth in PC12 cells. *PLOS ONE* 7:e43304. 10.1371/journal.pone.0043304 22916239 PMC3420912

[B27] Luikart B. W. Bensen A. L. Washburn E. K. Perederiy J. V. Su K. G. Li Y. (2011). MiR-132 mediates the integration of newborn neurons into the adult dentate gyrus. *PLOS ONE* 6:e19077. 10.1371/journal.pone.0019077 21611182 PMC3096628

[B28] Lungu G. Stoica G. Ambrus A. (2013). MicroRNA profiling and the role of microRNA-132 in neurodegeneration using a rat model. *Neurosci. Lett.* 553 153–158. 10.1016/j.neulet.2013.08.001 23973300

[B29] Magill S. T. Cambronne X. A. Luikart B. W. Lioy D. T. Leighton B. H. Westbrook G. L. (2010). MicroRNA-132 regulates dendritic growth and arborization of newborn neurons in the adult hippocampus. *Proc. Natl. Acad. Sci. U.S.A.* 107 20382–20387. 10.1073/pnas.1015691107 21059906 PMC2996687

[B30] Majer A. Medina S. J. Niu Y. Abrenica B. Manguiat K. J. Frost K. L. (2012). Early mechanisms of pathobiology are revealed by transcriptional temporal dynamics in hippocampal CA1 neurons of prion infected mice. *PLOS Pathog.* 8:e1003002. 10.1371/journal.ppat.1003002 23144617 PMC3493483

[B31] Miller B. H. Zeier Z. Xi L. Lanz T. A. Deng S. Strathmann J. (2012). MicroRNA-132 dysregulation in schizophrenia has implications for both neurodevelopment and adult brain function. *Proc. Natl. Acad. Sci. U.S.A.* 109 3125–3130. 10.1073/pnas.1113793109 22315408 PMC3286960

[B32] Min S. W. Cho S. H. Zhou Y. Schroeder S. Haroutunian V. Seeley W. W. (2010). Acetylation of tau inhibits its degradation and contributes to tauopathy. *Neuron* 67 953–966. 10.1016/j.neuron.2010.08.044 20869593 PMC3035103

[B33] Miyazaki Y. Li R. Rezk A. Misirliyan H. Moore C. Farooqi N. (2014). A novel MicroRNA-132-Surtuin-1 axis underlies aberrant B-cell cytokine regulation in patients with relapsing-remitting multiple sclerosis. *PLOS ONE* 9:e105421. 10.1371/journal.pone.0105421 25136908 PMC4138149

[B34] Nakahama T. Hanieh H. Nguyen N. T. Chinen I. Ripley B. Millrine D. (2013). Aryl hydrocarbon receptor-mediated induction of the microRNA-132/212 cluster promotes interleukin-17-producing T-helper cell differentiation. *Proc. Natl. Acad. Sci. U.S.A.* 10 11964–11969. 10.1073/pnas.1311087110 23818645 PMC3718186

[B35] Nudelman A. S. DiRocco D. P. Lambert T. J. Garelick M. G. Le J. Nathanson N. M. (2010). Neuronal activity rapidly induces transcription of the CREB-regulated microRNA-132, in vivo. *Hippocampus* 20 492–498. 10.1002/hipo.20646 19557767 PMC2847008

[B36] Numakawa T. Richards M. Adachi N. Kishi S. Kunugi H. Hashido K. (2011a). MicroRNA function and neurotrophin BDNF. *Neurochem. Int.* 59 551–558. 10.1016/j.neuint.2011.06.009 21723895

[B37] Numakawa T. Yamamoto N. Chiba S. Richards M. Ooshima Y. Kishi S. (2011b). Growth factors stimulate expression of neuronal and glial miR-132. *Neurosci. Lett.* 505 242–247. 10.1016/j.neulet.2011.10.025 22027176

[B38] Peng J. Omran A. Ashhab M. U. Kong H. Gan N. He F. (2013). Expression patterns of miR-124, miR-134, miR-132, and miR-21 in an immature rat model and children with mesial temporal lobe epilepsy. *J. Mol. Neurosci.* 50 291–297. 10.1007/s12031-013-9953-3 23315173

[B39] Qi Y. Zhu Z. Shi Z. Ge Y. Zhao K. Zhou M. (2014). Dysregulated microRNA expression in serum of non-vaccinated children with varicella. *Viruses* 6 1823–1836. 10.3390/v6041823 24759212 PMC4014722

[B40] Remenyi J. van-den Bosch M. W. Palygin O. Mistry R. B. McKenzie C. Macdonald A. (2013). MiR-132/212 knockout mice reveal roles for these miRNAs in regulating cortical synaptic transmission and plasticity. *PLOS ONE* 8:e62509. 10.1371/journal.pone.0062509 23658634 PMC3637221

[B41] Reschke C. R. Henshall D. C. (2015). MicroRNA and epilepsy. *Adv. Exp. Med. Biol.* 888 41–70. 10.1007/978-3-319-22671-2_4 26663178

[B42] Salta E. Lau P. Sala F. C. Coolen M. Bally-Cuif L. De-Strooper B. (2014). A self-organizing miR-132/Ctbp2 circuit regulates bimodal notch signals and glial progenitor fate choice during spinal cord maturation. *Dev. Cell* 30 k423–436. 10.1016/j.devcel.2014.07.006 25132384

[B43] Shaltiel G. Hanan M. Wolf Y. Barbash S. Kovalev E. Shoham S. (2013). Hippocampal microRNA-132 mediates stress-inducible cognitive deficits through its acetylcholinesterase target. *Brain Struct. Funct.* 218 59–72. 10.1007/s00429-011-0376-z 22246100 PMC3535403

[B44] Smith P. Y. Delay C. Girard J. Papon M. A. Planel E. Sergeant N. (2011). MicroRNA-132 loss is associated with tau exon 10 inclusion in progressive supranuclear palsy. *Hum. Mol. Genet.* 20 4016–4024. 10.1093/hmg/ddr330 21807765

[B45] Sun M. Yamashita T. Shang J. Liu N. Deguchi K. Feng J. (2015). Time-dependent profiles of microRNA expression induced by ischemic preconditioning in the gerbil hippocampus. *Cell Transplant.* 24 367–376. 10.3727/096368915X686869 25646661

[B46] Vo N. Klein M. E. Varlamova O. Keller D. M. Yamamoto T. Goodman R. H. (2005). A cAMP-response element binding protein-induced microRNA regulates neuronal morphogenesis. *Proc. Natl. Acad. Sci. U.S.A.* 102 k16426–16431. 10.1073/pnas.0508448102 16260724 PMC1283476

[B47] Wanet A. Tacheny A. Arnould T. Renard P. (2012). MiR-212/132 expression and functions: within and beyond the neuronal compartment. *Nucleic Acids Res.* 40 4742–4753. 10.1093/nar/gks151 22362752 PMC3367188

[B48] Wang R. Y. Phang R. Z. Hsu P. H. Wang W. H. Huang H. T. Liu I. Y. (2013). In Vivo knockdown of hippocampal miR-132 expression impairs memory acquisition of trace fear conditioning. *Hippocampus* 23 625–633. 10.1002/hipo.22123 23520022

[B49] Wayman G. A. Davare M. Ando H. Fortin D. Varlamova O. Cheng H. Y. (2008). An activity-regulated microRNA controls dendritic plasticity by down-regulating p250GAP. *Proc. Natl. Acad. Sci. U.S.A.* 105 9093–9098. 10.1073/pnas.0803072105 18577589 PMC2449370

[B50] Wong H. K. Veremeyko T. Patel N. Lemere C. A. Walsh D. M. Esau C. (2013). De-repression of FOXO3a death axis by microRNA-132 and -212 causes neuronal apoptosis in Alzheimer’s disease. *Hum. Mol. Genet.* 22 3077–3092. 10.1093/hmg/ddt164 23585551 PMC13387022

[B51] Xiang L. Ren Y. Cai H. Zhao W. Song Y. (2015). MicroRNA-132 aggravates epileptiform discharges via suppression of BDNF/TrkB signaling in cultured hippocampal neurons. *Brain Res.* 1622 484–495. 10.1016/j.brainres.2015.06.046 26168887

[B52] Xiao J. Li Y. Prandovszky E. Karuppagounder S. S. Talbot C. C. Jr. Dawson V. L. (2014). MicroRNA-132 dysregulation in *Toxoplasma gondii* infection has implications for dopamine signaling pathway. *Neuroscience* 268 128–138. 10.1016/j.neuroscience.2014.03.015 24657774 PMC4085776

[B53] Yang D. Li T. Wang Y. Tang Y. Cui H. Tang Y. (2012). MiR-132 regulates the differentiation of dopamine neurons by directly targeting Nurr1 expression. *J. Cell Sci.* 125 1673–1682. 10.1242/jcs.086421 22328530

[B54] Yang Y. C. Wei M. C. Huang T. C. (2012). Optimisation of an ultrasound-assisted extraction followed by RP-HPLC separation for the simultaneous determination of oleanolic acid, ursolic acid and oridonin content in *Rabdosia rubescens*. *Phytochem. Anal.* 23 627–636. 10.1002/pca.2365 22706975

[B55] Yao C. Shi X. Zhang Z. Zhou S. Qian T. Wang Y. (2016). Hypoxia-induced upregulation of miR-132 promotes schwann cell migration after sciatic nerve injury by targeting PRKAG3. *Mol. Neurobiol.* 53 5129–5139. 10.1007/s12035-015-9449-y 26399639

[B56] Yeung F. Hoberg J. E. Ramsey C. S. Keller M. D. Jones D. R. Frye R. A. (2004). Modulation of NF-kB-dependent transcription and cell survival by the SIRT1 deacetylase. *EMBO J.* 23 2369–2380. 10.1038/sj.emboj.7600244 15152190 PMC423286

[B57] Yi L. T. Li J. Liu B. B. Luo L. Liu Q. Geng D. (2014). BDNF-ERK-CREB signalling mediates the role of miR-132 in the regulation of the effects of oleanolic acid in male mice. *J. Psychiatry Neurosci.* 39 348–359. 10.1503/jpn.130169 25079084 PMC4160364

[B58] Yoshimura A. Numakawa T. Odaka H. Adachi N. Tamai Y. Kunugi H. (2016). Negative regulation of microRNA-132 in expression of synaptic proteins in neuronal differentiation of embryonic neural stem cells. *Neurochem. Int.* 97 26–33. 10.1016/j.neuint.2016.04.013 27131735

[B59] Yu H. C. Wu J. Zhang H. X. Zhang G. L. Sui J. Tong W. W. (2015). Alterations of miR-132 are novel diagnostic biomarkers in peripheral blood of schizophrenia patients. *Prog. Neuropsychopharmacol. Biol. Psychiatry* 63 23–29. 10.1016/j.pnpbp.2015.05.007 25985888

[B60] Zhang Z. Chang H. Li Y. Zhang T. Zou J. Zheng X. (2010). MicroRNAs: potential regulators involved in human anencephaly. *Int. J. Biochem. Cell Biol.* 42 367–374. 10.1016/j.biocel.2009.11.023 19962448

